# GP workforce sustainability to maximise effective and equitable patient care: a realist review

**DOI:** 10.3399/BJGP.2025.0061

**Published:** 2026-01-27

**Authors:** Emily Owen-Boukra, Bryan Burford, Tanya Cohen, Claire Duddy, Harry Dunn, Vacha Fadia, Claire Goodman, Cecily Henry, Elizabeth I Lamb, Margaret Ogden, Tim Rapley, Eliot L Rees, Nia Roberts, Etienne Royer-Gray, Gillian Vance, Geoff Wong, Sophie Park

**Affiliations:** 1 Nuffield Department of Primary Care Health Sciences, University of Oxford, Oxford, UK; 2 School of Medicine, Newcastle University, Newcastle upon Tyne, UK; 3 School of Clinical Medicine, University of Cambridge, Cambridge, UK; 4 Faculty of Medical Sciences, University College London, London, UK; 5 Centre for Research in Public Health and Community Care, University of Hertfordshire, Hatfield, UK; 6 Department of Social Work, Education and Community Wellbeing, Northumbria University, Newcastle upon Tyne, UK; 7 St George’s School of Health and Medical Sciences, City St George’s, University of London, London, UK; 8 Bodleian Health Care Libraries, University of Oxford, Oxford, UK; 9 Research Department of Primary Care and Population Health, University College London, London, UK

**Keywords:** equitable patient care, general practice, healthcare systems, personnel turnover, realist review, retention, workforce sustainability

## Abstract

**Background:**

UK and global primary care face significant GP workforce shortages. Much research focuses on individual-level factors such as wellbeing, resilience, and professional identity; however, less attention has been given to organisational- and system-level influences on GP work and workforce sustainability.

**Aim:**

To examine how general practice work and healthcare systems support GP workforce sustainability and effective, equitable patient care.

**Design and setting:**

This was a UK-focused realist review of empirical and grey literature. The search strategy encompassed six electronic databases.

**Method:**

The realist synthesis involved 1) finding existing theories, 2) searching for evidence, 3) selecting articles, 4) extracting data, and 5) synthesising evidence and drawing conclusions. Context–mechanism–outcome configurations were developed using extracted data, alongside input from patient and public contributors and stakeholders to iteratively refine the programme theory.

**Results:**

In total, 190 documents were included. Findings highlight the importance of meaningful work and engagement; relationships across individuals, organisations, and communities; and learning and development. Sustaining the GP workforce and delivering effective and equitable patient care require congruence between GPs’ core values and their work; cumulative-knowledge building; system agility; psychological safety; and direct human connections.

**Conclusion:**

Structures, policies, and relational connections within general practice are central for sustaining the GP workforce and enabling effective, equitable patient care. Collaboration among GPs, patients, and policymakers is essential. Future systems should prioritise personalised care, support meaning making, and protect GP autonomy to foster sustained engagement, expertise, and equity in care delivery.

## How this fits in

Global and UK primary care are experiencing a crisis because of GP workforce shortages. Previous UK GP workforce research has emphasised individual-level factors and solutions, including wellbeing, self-efficacy, training readiness, resilience, and professional identity shifts. This realist review examines the organisational- and system-level factors affecting GPs in their work and proposes recommendations to support a sustainable GP workforce and the delivery of effective and equitable patient care.

## Introduction

Improving healthcare access, quality, and efficiency in general practice requires multiple factors including the availability, expertise, and distribution of healthcare professionals.^1^ Global and UK primary care is experiencing a crisis characterised by substantial GP workforce shortages.^2–6^ UK GPs report low job satisfaction and high workload burden.^1^ Many GPs have left or are considering leaving their profession.^7–9^ The GP shortage crisis is critical to the affordability and sustainability of future healthcare systems and patient care. GPs provide safe, high-quality, holistic, and comprehensive care.^10,11^ They balance gate-keeping (for example, limiting patient medicalisation and investigation) and gate-opening (for example, advocacy activities), enhancing patient safety, health system efficiency, and health equity.^10,12–14^

Worldwide policies and strategies to address GP shortages and workload pressures include targeted funding schemes, investment in technology and infrastructure, and the integration of additional healthcare professionals (that is, non-GP staff introduced through workforce expansion initiatives, such as clinical pharmacists, physician associates, mental health practitioners, and social prescribers).^15–17^ Although these strategies have the potential to support workforce sustainability,^1,18^ there is limited evidence regarding the key causal factors or the effectiveness of specific approaches across diverse contexts.

The current review examines how GPs can be supported to flourish in their work, focusing on the organisational- and system-level characteristics that influence workforce sustainability. Previous research concerning the UK GP workforce has frequently framed challenges as individual-level ‘choices’, resulting in solutions that emphasise individual aspects, such as GP wellbeing, self-efficacy, training readiness, resilience, and professional identity.^5,19,20^ Campbell *et al* ^21^ and Sturmberg *et al* ^11^ emphasised the need to understand GP workforce sustainability within a broader social system, influenced by interpersonal and organisational dynamics. Guided by input from patient and public involvement (PPI) contributors and stakeholders, this review not only addressed the challenges but also examined system-level factors that either promote or hinder a sense of ‘joy’ in general practice, informing recommendations to sustain a workforce capable of delivering effective and equitable patient care.

## Method

Realist review is a theory-driven approach for synthesising diverse and complex evidence.^22,23^ It develops causal explanations of what works, for whom, and under what circumstances by identifying interactions between contexts (conditions), mechanisms (underlying causal forces), and outcomes (intended and unintended), forming context–mechanism–outcome configurations (CMOCs). For example, when GPs can allocate time to aspects of their practice they consider meaningful (context), they are more engaged in their work (outcome), because they experience a sense of congruence between their core values and the nature of work (mechanism). These configurations iteratively shape the development of a programme theory, an overarching explanation of the topic and research question(s). Realist reviews use diverse literature to understand complex interventions from multiple perspectives,^23^ incorporating experiential expertise through PPI and stakeholders, enhancing the research team’s critical engagement during data analysis.

This review, registered on PROSPERO (CRD42023395583), adheres to the RAMESES (Realist and Meta-narrative Evidence Synthesis: Evolving Standards) quality and reporting standards.^24,25^ Full methodological details, including the authors’ initial programme theory (IPT), are available in the published protocol.^26^

IPT development involved a scoping review of the literature alongside discussions with PPI members and stakeholders, including GPs. The stakeholder group also included a medical student, a hospital clinician, and a physician associate, contributing diverse perspectives from primary care and the wider healthcare workforce. PPI members offered insights into aspects of general practice they perceived as supporting effective and equitable patient care, while stakeholders reflected on factors that contribute to, or undermine, joy in GP work.^26^

The IPT focused on long-term challenges related to GP recruitment and retention, recent systemic changes, and the potential role of joy and meaning in GP work. Initial discussions with PPI and stakeholders identified key challenges, while also highlighting positive factors for further exploration, such as meaningful interactions, connection, kindness, and collaboration. In response to this input, this study used the terms joy, flourishing, and thriving interchangeably to describe positive and sustaining experiences in GP work, those that support professional engagement, a sense of meaning, and long-term sustainability.^27^ As the review progressed, the authors developed 18 CMOCs (see Supplementary Table S2 for CMOCs and supporting evidence), drawing on literature and PPI/stakeholder insights. Related CMOCs were synthesised, forming the analytical foundation for refining the current study’s programme theory into three overarching, interrelated categories that structure the final synthesis.

In April 2023, formal searches were conducted by the fourth author (Supplementary Information S1), using six electronic databases: Medline, Embase, PsycINFO, CINAHL, Health Management Information Consortium (HMIC), and Web of Science Core Collection (SCIE, SSCI, AHCI). These databases were selected to capture both academic and grey literature across health and interdisciplinary fields. Grey literature was also identified through HMIC and targeted searches of policy reports, editorials, letters, and non-peer-reviewed sources. These searches initially yielded 1463 documents.

To ensure the review remained current, updated searches were conducted in April 2025 by the thirteenth author, identifying an additional 23 relevant documents published or updated since April 2023, bringing the total number of included documents to 190. The inclusion and exclusion criteria are summarised in Box 1.

Documents were assessed for relevance by the first, second, ninth, and twelfth authors for the initial set, and by the fifth author for the updated set. Relevance was defined as containing data related to context, mechanisms, and outcomes and contributing to the refinement of the IPT. Rigour was assessed based on the credibility, plausibility, and trustworthiness of the methods used to generate the relevant data.^23,24^

Box 1. **Inclusion and exclusion criteria**

**Inclusion**

- Publications from 2013 onward
- UK publication
- GP or UK general practice focus
- Content pertains to relationship between GPs and work, or meaning attributed to work

**Exclusion**

- Only trainee participants
- Recruitment only (that is, nothing about retention)
- No rich or detailed information relevant to initial programme theory

Programme theory refinement involved synthesising the literature and PPI/stakeholder consultations to scrutinise data sources and formulate explanatory CMOCs. Document characteristics were extracted into a Microsoft Word document (Supplementary Table S1). Relevant data were interpreted, coded, and synthesised using a realist logic of analysis. CMOCs were developed using narrative summaries and illustrative quotations. Emerging analytical findings and CMOCs were critically discussed by the research team, including PPI co-applicants, to support CMOC modification, expansion, and refinement (see Supplementary Table S2). These processes led to the inclusion of 190 documents and informed the synthesis of findings across three overarching categories.

## Results

**Figure 1.**
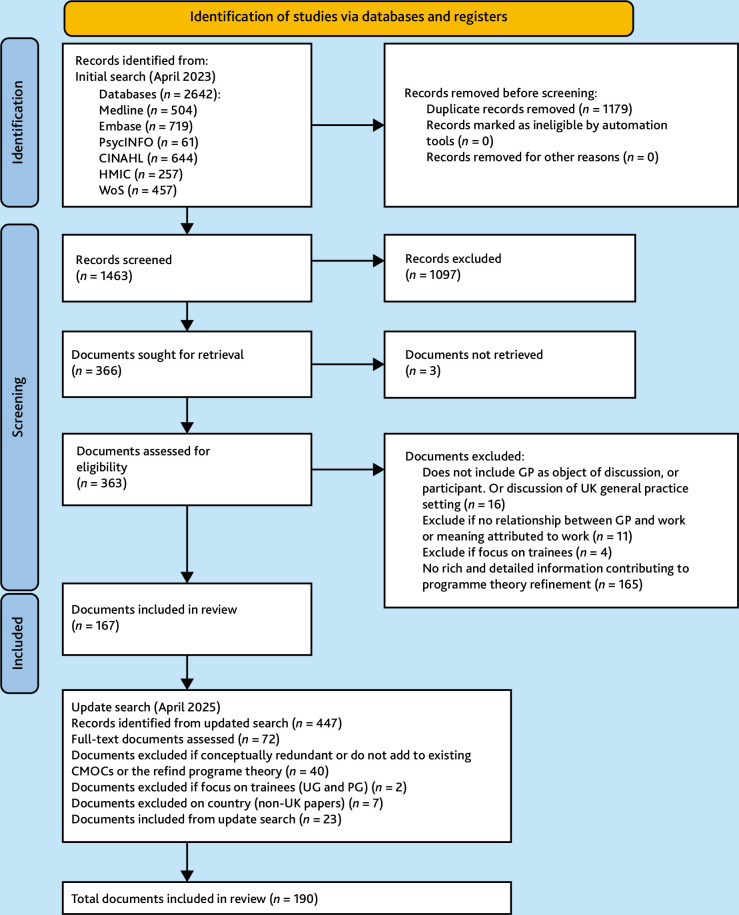
PRISMA summary of searching and selection processes. CMOC = context–mechanism–outcome configuration. PG = postgraduate. UG = Undergraduate. WoS = Web of Science.

A total of 190 documents published between 2013 and 2025 were identified for inclusion (Figure 1), comprising 122 published research articles, three conference abstracts, and 65 other sources (such as policy reports, guidance articles, editorials, and books). Supplementary Table S1 presents the main characteristics of the included documents.

Findings are organised into three overarching and interrelated categories:

- meaningful work and engagement (including meaning making; mutual care; balancing workload and resources; and hidden administrative work);
- relationships across individuals, organisations, and communities (including knowledge accumulation: long-term patient–GP relationships; connection-rich contexts; relationships with additional healthcare professionals); and
- learning and development (focused on enabling cultures and organisations).

Each category is influenced by opportunities for direct interactions and connections within work.

Although the authors’ focus was on organisational- and system-level influences, the authors’ realist lens made it possible to examine how these broader structures and ideologies, such as how work is organised and what is valued as 'good work', shape individual-level experiences and responses, including the meaning GPs derive from their work. These responses are conceptualised as mechanisms or outcomes within the CMOCs.

### Meaningful work and engagement

Contributing and mitigating factors were identified across the subcategories of meaning making; mutual care; balancing workload and resources; and hidden administrative work.

#### Meaning making

For many GPs, it is essential that their work is purposeful, significant, and aligns with their core values.^11,28–31^ When GPs are able to allocate time to aspects of their practice they consider meaningful (for example, direct doctor–patient interactions, or administrative tasks pertaining to known patients), they are more engaged in their work because they experience a sense of congruence or alignment between their core values and the nature of work (CMOC1).^5,28–30,32–37^ The experiences and perceptions of meaningful work varied among GPs. GPs derive unity, purpose, and meaning through long-term therapeutic relationships with patients and families, in addition to performing patient advocacy, health promotion work, community participation, and improving local service provision.^5,11,28,31,35,37–46^

GPs find intellectual stimulation in managing ill-defined illnesses, chronic complex multimorbidity, and accurate diagnoses.^5,28,37,39,46–49^ GPs indicated the highest level of satisfaction and meaning when experiencing feelings of competency and mastery,^5,11,39^ and from consultations in which they perceived their personal contributions to have resulted in successful patient outcomes.^28,37^ The act of mentoring and teaching medical students and junior colleagues through involvement in undergraduate and postgraduate training schemes is also particularly meaningful.^28,32,33,46,50^ Many GPs value the opportunity to contribute to the professional development of others and to reciprocate the support they received during their own training.^37^

GPs encounter multiple challenges that hinder their engagement in meaningful work. Addressing these challenges requires the volume and nature of GP work to be proportionate to the available time, resources, and professional autonomy. When this balance is disrupted, such as when administrative responsibilities overshadow clinical care and direct patient interactions, it becomes difficult for GPs to derive meaning from their roles. In contexts where policies and guidelines emphasise compartmentalised (such as, single-disease management) and/or commodified (such as, payment or reward-driven) models of care, the doctor–patient relationship can become distorted. GPs may feel pressure to reduce opportunities for therapeutic connections, situated knowledge, and continuity of care (CMOC2).^37,51,52^ This can compromise GPs’ ability to address diverse patient needs, disproportionately affecting the patients who are the most vulnerable and socioeconomically disadvantaged.^19,29,35,37,40,50^ Many GPs find aspects of their work intellectually stimulating, fulfilling, and meaningful. However, most report pressure to balance patient/carer needs against target-driven accountability, stringent bureaucratic monitoring, and a standards-driven reward system.^5,29,47,50,52–55^ Although larger practice sizes do not inhibit meaningful work, certain common at-scale organisational approaches (for example, task delegation) can disintegrate and/or compartmentalise work (for example, repeat prescribing, filing results, and acute provision of clinical care), minimising personal continuity and reducing opportunities for meaningful connections.^56^

#### Mutual care

Media and policy attention has recently oriented general practice systems towards prioritising rapid access, often at the expense of key elements that underpin sustainable, meaningful, and equitable care.^19,57^ A critical insight shared by the PPI co-applicants, and supported in the literature, was the concept of reciprocal care (CMOC3): the understanding that caring relationships in general practice involve mutual recognition, respect, and emotional investment from both GPs and patients. As one PPI co-applicant asked, *‘What about patient care for their GP?’* Current policies tend to position ‘patient demand’ as a fixed entity (and often expanding or overwhelming). The current study’s analysis, however, suggests that demand is dynamic: constructed and negotiated through social interactions between patients, GPs, and practices. When systems become depersonalised, for example, by prioritising the speed of triage/access, these relational dynamics may be disrupted.^19,29^ Patients may be (re)positioned as consumers and experience reduced personal connections with their GP or practice.

This erosion of relationship-based care reduces opportunities for GPs to engage in ‘holding work’ that encompasses the emotional, relational, and advocacy labour involved in navigating uncertainty, providing continuity, and supporting patients in managing concerns over time.^58,59^ Similar practices are evident in the work of primary care link workers.^60^ Without a sense of connection, patients may feel overwhelmed and uncertain, potentially resulting in more frequent or urgent service use.

When familiarity with a practitioner exists, patients are more likely to trust their GP and feel cared for.^31,43^ This familiarity facilitates opportunities for collaborative preparation or pre-emptive planning regarding ‘what to look out for’ or ‘when to worry’, as well as a mutual understanding of temporal expectations (such as previous patterns for these symptoms and/or this patient, plus likely duration self-limiting, short or long term). Patients can reciprocate care and empathy for their practitioners and, when safe, may moderate their help-seeking behaviours where they feel they have adequate information and trust their GP, minimising a sense of crisis, perceived urgency of healthcare needs, and lowering the likelihood of complaints.^19,31,43,46^

Health systems shaped by transactional logics, structural inequities, and technocratic priorities often favour patients who are confident, articulate, and health literate, as they are better equipped to command attention and navigate complex institutional processes.^61^ Conversely, patients facing socioeconomic disadvantage, language or communication barriers, or complex life challenges may find themselves further removed from general practice support, thereby exacerbating existing inequalities.^19,62^ For GPs, these system dynamics can constrain relational care, limit continuity, and pose challenges to delivering effective and equitable care. Policies and service models that conceptualise healthcare access as transactional or problem-focused frequently presuppose that patients present with clearly defined, pre-formed issues.^63^ This perspective overlooks the critical role of ‘problem-setting’, a fundamental component of GP–patient interactions that facilitates collaborative identification and prioritisation of concerns. Supporting this process enables more sustainable, person-centred care and helps reduce experiences of disempowerment, apprehension, and hopelessness.^38^

#### Balancing workload and resources

Workload (for example, demand, nature, and quantity) and resource imbalances (for example, finance, peer, and organisational support), can impede meaningful work (CMOC4).^5,19,29,32,34,38,45,50,55,64–68^ When GPs experience consistently high workloads with limited resources and respite, they are at risk of burnout, as they feel out of control and overwhelmed (CMOC5).^5,16,19,29,31,38,64–66^ Despite the assumption that remote work enhances efficiency, the transformation from face-to-face to remote consulting can paradoxically increase GP workload because of increased patient access, expectation/demands, increased negotiation of risk, and limited opportunities for GPs to schedule breaks and/or time with colleagues (CMOC6).^19,69,70^

#### Hidden administrative work

Numerous studies identify administrative work as problematic.^19,29,32,47,52,55,66,71^ It is frequently categorised as ‘hidden’ or ‘invisible’, reflecting a lack of allocated, explicit, or scheduled time (in contrast to a hospital consultant who might have a combination of clinical and administrative sessions).^50^ Administrative work was more acceptable when it could be attributed meaning, such as when it was requested by the GP, or involved reviewing the results and considering management options for a known patient. When work organisation resulted in GPs seeing fewer patients (for example, owing to delegation to additional healthcare professionals) to perform more administrative tasks and/or these tasks became disconnected from known patients, administrative work was perceived as more challenging, risky, and burdensome.^19,66^ However, in contexts where direct human connections and face-to-face interactions with patients and colleagues are present, paperwork/administrative work can be perceived as a means to a larger end and become an accepted part of GPs’ work.^32,46^

### Relationships across individuals, organisations, and communities

#### Knowledge accumulation: long-term patient–GP relationships

Irrespective of educational, social, and cultural context, some assume that learning is acontextual.^72^ However, this review demonstrates the centrality of situated learning and expertise.^72^ By nurturing situated learning in general practice (emphasising the importance of social interactions with patients and peers, and conceptualising learning and knowledge accumulation as a dynamic process), GPs described more meaningful work.^11,28,29,31,40–42^ For instance, when systems and structures support continuity and are designed to accumulate knowledge about individuals (people) and communities (place), this facilitates more appropriate patient care, as GPs can draw on and adapt their deeper and expanded understanding in GP–patient and GP–peer encounters (CMOC7). ^11,16,28,31,32,37,38,40,73^ In such contexts, some GPs described developing a deep contextual understanding of patients’ personal circumstances, which informed and shaped their clinical decision making.^38^ Familiarity with the local area and broad knowledge of community needs and assets further enhanced how and when GPs applied this contextual knowledge to guide care and advocate at both individual and community levels.^11,28,38,43^

The growth and implementation of cumulative knowledge can flourish through interactions with patients and peers (both within and beyond the institution), cultivating knowledge of local systems and community preferences.^28,37,40,55,57,73^ This facilitates opportunities for patient partnership and advocacy (‘gate-opening’), as well as the development and use of adaptive expertise to address specific circumstances in a patient-centred manner.^31,38,57,74^ In contexts where GPs, patients, and peers have opportunities to shape and co-create management plans and potential solutions, this may promote patient and practitioner enablement and satisfaction, resulting in higher levels of peer and patient trust, and improved self-management capacity (CMOC8).^31,37,38,40^ From a patient’s perspective, mutual trust and respect were deemed essential.^31^

#### Connection-rich contexts

Direct interactions and connections, such as a phone call with a colleague or patient, or the filing and actioning of results for a known patient, create opportunities for meaningful practice, the development and application of cumulative knowledge, and agile, adaptable approaches to personalised care.^38,40^ These interactions facilitate meaningful engagement and psychological safety for GPs as they navigate risk, uncertainty, and ambiguity in practice (CMOC9). Excessive shifts towards indirect interactions (for example, managing substantial risk through the supervision of additional healthcare professional–patient interactions, or reviewing results for unfamiliar patients and making disembodied decisions without direct patient interaction or contextual understanding) can increase both patient safety risks and work-related risks for GPs.^19,56^ These dynamics may contribute to diminished GP experiences of connection, engagement, professional satisfaction, and increased risk of burnout.^70^

GPs support many patients presenting with undifferentiated illnesses, characterised by vague, non-specific, or not yet diagnosable symptoms, as well as those with complex needs and multiple conditions. In such contexts, protocol-driven or standardised care may be less safe or desirable for patients, potentially leading to inappropriate and additional (costly) demands on healthcare services.^14^

Managing these compromises and adaptations requires complex, dynamic negotiation of risk with patients. GPs mitigate these risks not by standardising, but by rapidly exchanging, adapting, and co-developing management plans through direct interaction. When interactions become indirect, the navigation of uncertainty can become overwhelming and unmanageable, having an impact on both patient care and GP wellbeing.^19^ In contexts where GPs experience reduced human connections with colleagues and patients this can contribute to isolation, reduced sensitivity, and motivation because work becomes depersonalised (CMOC10).^29,34,35,40,45,47,54,55,75^

#### Relationships with additional healthcare professionals

Effective collaborative relationships with additional healthcare professionals may contribute to meaningful work.^53,76–78^ However, the reviewed literature identifies a potential paradox in delegating work to additional healthcare professionals. Managing risk and uncertainty can be more complex when interactions between patients and colleagues are indirect.^79–81^ Unlike secondary care, where protocolised management of specific conditions may be effective, GPs frequently support patients with undifferentiated, multiple, and complex conditions. This has significant implications for the support, time, and nature of supervision that GPs must provide to additional healthcare professionals working alongside them to ensure effective and equitable patient care.^82^ When tasks typically undertaken by the GP are delegated to others, this can allow GPs to focus on more complex patients but can increase ‘indirect’ care (for example, supervising or holding responsibility for another’s work and management), and may erode continuity of care (CMOC11.1).^65,79,82^ Triaging ‘simpler’ or ‘appropriate’ patients for staff can be challenging in the context of undifferentiated illnesses. However, when patients with potentially less complex problems are seen by practice members other than the GP, this can result in GPs managing sustained high levels of clinical complexity, leading to relentless emotional and cognitive load (CMOC11.2).^83^ In contexts where there is a discrepancy between service-learning expectations and the needs of additional healthcare professional roles in general practice, successful integration can be challenging unless sufficient time and resources are allocated for GPs to provide generalist training to colleagues (CMOC12).

Importantly, in contexts where adequate infrastructure and support are present, and where additional roles complement rather than replace the core GP–patient interaction, some GPs reported that the specialist knowledge provided by roles such as clinical pharmacists enhanced decision making and improved patient care.^78^ This aligns with arguments for maintaining GP expertise early in the patient journey, with other roles contributing after the GP has helped frame and clarify the patient’s problem to ensure safe and effective care.^63^

### Learning and development

#### Enabling cultures and organisations

Regular interactions with colleagues, as well as established systems and routines for knowledge exchange, can enhance GPs’ sense of connection, adaptability, and coping mechanisms, thereby informing their clinical expertise and patient care (CMOC13).^28,32,33,41,45,65,84,85^ Such interactions include opportunities for informal engagement and peer support, including group practice meetings, team ‘huddles’, mentoring systems, coffee breaks, dinners, and quality circles (small groups of professionals who meet regularly to reflect on and improve practice). These interactions may foster a sense of community among GPs, which, in turn, facilitates their learning and flourishing (growth, development, and thriving) (CMOC14).^34,45,68,86,87^ Additional examples include personal, direct, and/or frequent exchanges between GPs and colleagues in secondary or community care. Such interactions can enhance patient safety and reduce fragmentation of care by strengthening local knowledge, mutual understanding, and coordination among healthcare professionals (CMOC15).^28,31,34,38,88–90^

A climate of psychological safety is widely recognised as important for facilitating learning and regular interactions among colleagues.^32,36,55,64,71,91,92^ When present (such as through supportive organisational practices and patient trust), GPs are better equipped to use their expertise to support patient care amid uncertainty (CMOC16). Trust is a crucial component of psychological safety, particularly in general practice, where clinicians routinely encounter risk and diagnostic uncertainty.^11,79,80^ Although guidelines can be beneficial, rigid expectations that all clinical work must be protocolised may leave GPs feeling disempowered. Such expectations restrict their autonomy and authenticity in providing relevant, patient-centred care, potentially leading to disengagement from their work.^11,32,33,55^ One unintended consequence of protocol overreliance is an increasing discrepancy between ‘imagined work’, conceptualised as a series of discrete, single-disease management pathways, and the reality of patient care. In practice, GPs often navigate complex, interrelated issues that span multiple biomedical domains and are deeply entwined with broader social or ecological factors, such as housing insecurity, psychosocial stressors, or experiences of domestic violence.

## Discussion

### Summary

Numerous synergies exist between achieving effective and equitable patient care and ensuring a sustainable future GP workforce. Social interactions within general practice, particularly those between GPs and patients, shape healthcare delivery, influence the proportionate support necessary for universal care, and have an impact on GP job satisfaction and workforce sustainability.^36,75,93^ GP work and patient engagement are dynamic and socially negotiated processes. Chronic underinvestment in UK general practice has led to overwhelming workloads and understaffing, negatively affecting workforce morale, particularly in areas of socioeconomic deprivation.^35,51,53^ Increased investment in the GP workforce is therefore crucial and should be proportionate to the volume and complexity of NHS care delivered in this setting.

It is not inevitable that modern general practice must involve impersonal patient interactions, excessive documentation, and relentless triage at the expense of meaningful patient care. Contrary to common assumptions,^94,95^ technology does not inherently improve efficiency in managing the complex, undifferentiated workload typical of general practice. The current study’s findings reconceptualise the nature of current ‘GP workforce problems’. Rather than simply expanding access to any practitioner or incorporating additional triage and indirect supervision, the authors of the current study argue that system designs should prioritise GP–patient connection, which is essential for navigating risk, managing ambiguity, and delivering equitable care.^35,69,75^ Although there is no universal approach, this review offers core principles, which, when applied in practice, can support joy in GP work and the provision of effective and equitable patient care.

Organisational and financial models are critical for GP workforce sustainability. Financial incentives influence both the nature of work and GP engagement in general practice.^37,51,52,96^ Although meaning making is not solely determined by financial factors, these models significantly shape team composition, the prioritisation of patient needs, and the structuring of appointment systems. Consequently, they affect the opportunities and challenges that GPs face both within and beyond clinical work.^55^ Previous research^97–99^ has highlighted the importance of prioritising collective outcome goals over individual financial incentives to foster collaboration and service integration. This review also demonstrates how the commodification of care can affect GP–patient interactions, often prioritising short-term financial objectives over meaningful discussions or spontaneous engagement.^31,37,52,54^

One critical area warranting attention is the operationalisation of roles, delegation, and supervision within multidisciplinary teams. This review’s findings highlight that, although delegating tasks to additional healthcare professionals can enable GPs to focus on more complex care, it often results in indirect responsibility, increasing both clinical risk and emotional burden.^79,83^ This is particularly challenging when GPs are required to hold clinical risk through indirect patient contacts, review results and make decisions for unfamiliar patients, or provide supervision under constrained time and resource conditions. For instance, GPs reported difficulty making safe decisions without understanding the context of a test request or the patient’s clinical history, which reduced professional satisfaction and heightened the risk of error.^19^ In addition, routine triage of patients with less complex cases to other team members may inadvertently have an impact on workforce sustainability by tipping the balance towards GPs predominantly managing complex, uncertain, or emotionally demanding presentations. Sustaining this intensity of work is incompatible with the constraints of the current 10-min appointment model and necessitates a fundamental redesign of consultation structures and workload planning.

To address these challenges, future systems should provide protected time for supervision, facilitate direct communication and continuity within teams, and ensure appropriate training and support for additional healthcare professionals.^73,78^ Effective delegation is more than a redistribution of tasks; it requires maintaining safe, coordinated, and learning-oriented care. To achieve this, delegation should occur at an appropriate stage in the patient journey, typically after a GP has assessed and problematised the case, ensuring that additional roles complement, rather than substitute, the core GP–patient interaction. These system-level supports are essential for delivering equitable patient care, ensuring that all patients receive high-quality, contextualised attention and care, regardless of their complexity.

Based on the findings of this review, alongside extensive stakeholder and patient engagement, the authors propose the following priorities for the organisation and delivery of general practice:

- support GPs in tolerating and negotiating risk through the nature and timing of patient and peer interactions;
- enable flexibility and agility to deliver personalised care; and
- empower GPs to work collaboratively with patients to align person-centred values with work requirements and activities.

These priorities can be achieved by focusing on three interconnected areas:

- meaningful work and engagement;
- relationships across individuals, organisations, and communities; and
- learning and development.

The authors’ refined programme theory summarises the review findings (Box 2). This theory integrates the CMOCs developed through the review to provide a cumulative synthesis. It is intended to inform future implementation strategies and guide policies and practices aimed at supporting a sustainable general practice workforce.

**Box 2.** Refined programme theory

Facilitators	
Meaningful work and engagement	Congruence between GPs’ core values and the nature of their work Opportunities for reciprocal care and mutual acts of compassion Balance work demands and available resources (for example, appropriate consultation time) Support GP roles as advocates and enable GP–patient agency Recognise and enable intellectual stimulation in GP work (for example, agile and flexible expertise to personalise/contextualise care)
Relationships across individuals, organisations, and communities	Connection-rich contexts (direct interactions and connections within work activities) Cultivate and use cumulative knowledge (regarding local people and place) to inform the organisation and delivery of care Facilitate direct connections and cross-disciplinary learning opportunities between peers and organisations Enable informal learning, engagement, and peer support
Learning and development	Climate of psychological safety that enables opportunities for care and ongoing practice-based learning with patients and peers Embed spaces for learning and exchanging cumulative knowledge into learning structures and systems Promote enabling cultures and dynamic learning systems that support effective delegation, structured supervision, and shared management of clinical complexity

Barriers	
Depersonalisation and commodification of GP work Lack of recognition/planning to support potential disconnection and additional work inherent in remote consulting Paradox of delegation to supervise and support additional healthcare professionals	

### Strengths and limitations

This realist review represents, to the authors’ knowledge, the first exploration of GP workforce sustainability, enhancing understanding of the factors that maintain and sustain GPs, addressing challenges, and offering recommendations for future primary care. The current research extends previous individual-oriented perspectives to a systemic view of organisational characteristics and the role of the social environment. The documents included encompass a diverse range of materials, including grey literature, conference materials, and policy reports, maximising the breadth and depth of the review. To enhance transferability, this study examined documents on GP workforce sustainability across various geographic settings. Through collaborative discussions and reflective dialogue with PPI and stakeholder groups, the authors verified the approach, expanded analytical possibilities, and ensured recommendations were relevant and applicable to policy, practice, and patient care. Although the current review is highly relevant to current and emerging policy developments, including the NHS Long Term Plan^94^ ^95^, it is limited by the evidence available at the time of the final update searches. Given the rapid evolution of the healthcare landscape, new literature relevant to GP workforce sustainability may continue to emerge. Nevertheless, the current study’s synthesis provides a robust foundation to inform future policy and practice.

### Comparison with existing literature

Prior UK GP workforce studies have predominantly focused on individual-level factors, such as GP wellbeing, self-efficacy, training readiness, resilience, and professional identity.^5,19^ This review presents a broader organisational- and system-level analysis of the factors that facilitate or impede GP workforce sustainability. The Institute for Healthcare Improvement’s recently published ‘joy in work’ framework^100^ aligns with the current review’s focus on promoting joy in general practice. Recommendations were developed (see Box 2) to guide future efforts in ensuring GP workforce sustainability and delivering effective and equitable patient care.

### Implications for research and practice

The development of a sustainable GP workforce to deliver effective and equitable patient care, as outlined in the NHS Long Term Workforce Plan,^94,101^ requires examination of the interrelationship between systemic and individual factors, and how these shape the nature of GP work and patient care. Persistent pressures pose the risk of fragmenting, compartmentalising, or partially privatising aspects of NHS general practice.^102^ Nevertheless, general practice is widely acknowledged for its clinical and economic value as the cornerstone of UK primary care. This underscores the need for sustainable investment and a long-term collaborative strategy to support the workforce and safeguard the future of general practice. Alignment with meaning making requires organisational and financial support to maximise sustained and direct connections with patients, peers, and communities, thereby fostering ongoing learning systems and cumulative knowledge development. This long-term approach enables GPs and patients to shape and maximise the forward planning of contacts, rather than treating each encounter and practice interaction as a discrete entity.

## Data

All data underpinning the findings of this realist review are provided in the online supplement, including data extraction tables, context–mechanism–outcome configurations (CMOCs), and supporting evidence derived from the included literature.
